# Evolution of CT Findings and Lung Residue in Patients with COVID-19 Pneumonia: Quantitative Analysis of the Disease with a Computer Automatic Tool

**DOI:** 10.3390/jpm11070641

**Published:** 2021-07-06

**Authors:** Roberto Grassi, Salvatore Cappabianca, Fabrizio Urraro, Vincenza Granata, Giuliana Giacobbe, Simona Magliocchetti, Diletta Cozzi, Roberta Fusco, Roberta Galdiero, Carmine Picone, Maria Paola Belfiore, Alfonso Reginelli, Umberto Atripaldi, Ornella Picascia, Michele Coppola, Elio Bignardi, Roberta Grassi, Vittorio Miele

**Affiliations:** 1 Division of Radiodiagnostic, Università degli Studi della Campania Luigi Vanvitelli, 80138 Naples, Italy; roberto.grassi@unicampania.it (R.G.); salvatore.cappabianca@unicampania.it (S.C.); fabrizio.urraro@unicampania.it (F.U.); giuliana.giacobbe@unicampania.it (G.G.); simona.magliocchetti@unicampania.it (S.M.); mariapaola.belfiore@unicampania.it (M.P.B.); alfonso.reginelli@unicampania.it (A.R.); umberto.atripaldi@studenti.unicampania.it (U.A.); ornella.picascia@studenti.unicampania.it (O.P.); robertagrassi89@gmail.com (R.G.); 2 Italian Society of Medical and Interventional Radiology (SIRM), SIRM Foundation, 20122 Milan, Italy; 3 Radiology Division, Istituto Nazionale Tumori IRCCS Fondazione Pascale—IRCCS di Napoli, 80131 Naples, Italy; r.galdiero@istitutotumori.na.it (R.G.); c.picone@istitutotumori.na.it (C.P.); 4 Division of Radiodiagnostic, Azienda Ospedaliero—Universitaria Careggi, 50139 Florence, Italy; dilettacozzi@gmail.com (D.C.); vmiele@sirm.org (V.M.); 5 Medical Oncology Division, Igea SpA, 80013 Naples, Italy; r.fusco@igeamedical.com; 6 Diagnostic Imaging Unit, “Azienda Ospedaliera dei Colli”—Ospedale Monaldi, 80131 Naples, Italy; michele.coppola@ospedalideicolli.it (M.C.); elio.bignardi@ospedalideicolli.it (E.B.)

**Keywords:** COVID-19, computed tomography, computer aided quantification

## Abstract

Purpose: the purpose of this study was to assess the evolution of computed tomography (CT) findings and lung residue in patients with COVID-19 pneumonia, via quantified evaluation of the disease, using a computer aided tool. Materials and methods: we retrospectively evaluated 341 CT examinations of 140 patients (68 years of median age) infected with COVID-19 (confirmed by real-time reverse transcriptase polymerase chain reaction (RT-PCR)), who were hospitalized, and who received clinical and CT examinations. All CTs were evaluated by two expert radiologists, in consensus, at the same reading session, using a computer-aided tool for quantification of the pulmonary disease. The parameters obtained using the computer tool included the healthy residual parenchyma, ground glass opacity, consolidation, and total lung volume. Results: statistically significant differences (*p* value ≤ 0.05) were found among quantified volumes of healthy residual parenchyma, ground glass opacity (GGO), consolidation, and total lung volume, considering different clinical conditions (stable, improved, and worsened). Statistically significant differences were found among quantified volumes for healthy residual parenchyma, GGO, and consolidation (*p* value ≤ 0.05) between dead patients and discharged patients. CT was not performed on cadavers; the death was an outcome, which was retrospectively included to differentiate findings of patients who survived vs. patients who died during hospitalization. Among discharged patients, complete disease resolutions on CT scans were observed in 62/129 patients with lung disease involvement ≤5%; lung disease involvement from 5% to 15% was found in 40/129 patients, while 27/129 patients had lung disease involvement between 16 and 30%. Moreover, 8–21 days (after hospital admission) was an “advanced period” with the most severe lung disease involvement. After the extent of involvement started to decrease—particularly after 21 days—the absorption was more obvious. Conclusions: a complete disease resolution on chest CT scans was observed in 48.1% of discharged patients using a computer-aided tool to quantify the GGO and consolidation volumes; after 16 days of hospital admission, the abnormalities identified by chest CT began to improve; in particular, the absorption was more obvious after 21 days.

## 1. Introduction

The spread of severe acute respiratory syndrome coronavirus 2 (SARS-CoV-2) has already assumed pandemic proportions, affecting over 100 countries in few weeks [1,2].

Currently, the “gold standard” for diagnosis of COVID-19 infection is a real-time reverse transcriptase polymerase chain reaction (RT-PCR) amplification of the viral DNA. However, radiological imaging is of great significance in the surveillance of COVID-19 infection [3,4,5]. Recent studies have demonstrated that CT findings of COVID-19 pneumonia show ground glass opacity (GGO) with surrounding consolidation, with bilateral involvement, peripheral distribution, and multi-lobar distribution [3,4,5,6,7].

However, the consolidation, or GGO with consolidation, increased, and reticular was observed in the later stages (scan > 1 week after symptom onset), this represents the conversion of findings from GGO to consolidation, and an increase in the reticulation pattern in affected lung parenchyma. CT features had rapid sever changes, from focal unilateral pulmonary parenchyma to diffuse bilateral GGO, or GGO with consolidation, within 1–3 weeks [6,7]. Although several studies have described the CT imaging features of COVID-19 pneumonia, so far, there is a lack of large-sample CT imaging studies and follow-up observations [8,9,10,11,12,13,14,15,16,17,18,19,20].

CT investigation in patients with suspected COVID-19 pneumonia involves the use of high-resolution techniques. Artificial intelligence (AI) software for quantification of pneumonia lesions has been employed to integrate CT diagnosis [15,16]. Computer software could be useful to categorize the disease into different severities, with quantitative, objective assessments of the extent of the lesions [17,18,19,20]. Computer tools have recently been proposed for the recognition of lung lesions (from COVID-19) on CT examinations [21,22,23]. However, many of them are not recognized as medical devices nor do they have the CE marking.

To the best of our knowledge, no study in the literature reports on the temporal changes of CT findings, using an automatic tool to quantify the abnormality in lung parenchyma, due to COVID-19 pneumonia, in a large dataset of patients.

We investigate the use of a computer-aided tool in order to quantify the abnormalities visible on chest CT images in patients with COVID-19 pneumonia.

The aim of this study was to assess the evolution of CT findings and lung residue in patients with COVID-19 pneumonia, performing quantitative analysis of the disease with the commercially available system.

## 2. Methods

### 2.1. Patient Characteristics

This retrospective study included patients enrolled by “Hospital of Colli (Monaldi-Cotugno-CTO)” in Naples. In relation to the ongoing epidemic emergency, the institutional local review boards gave up written informed consent for this retrospective study that evaluated anonymized data and involved no potential risk to patients. The population included 140 patients (50 women and 90 men; 68 years of median age—range, 25–92 years) subjected to the nucleic acid amplification test of the respiratory tract or blood specimens, using a reverse transcription real-time fluorescence polymerase chain reaction test, for suspicion of COVID-19, between 2 March 2020 and 5 May 2020. The virus investigation for etiological diagnosis was executed by the current gold standard test. All patients with a positive RT-PCR test at hospital admission and with respiratory distress were hospitalized and followed-up. The clinical evolution of the disease was subdivided in stable, improved, and worsened. The parameters considered took into account the fever (≤37.3, 37.4–38.0, >38.0) and the breathing with SpO2 value in ambient air, and the ratio PaO2/FiO2 (mild >200 up to 300 mm Hg; moderate >100 and ≤200 mm Hg, severe ≤100 mm Hg). The following laboratory parameters were assessed: white blood cells (Lymphopenia, leukopenia), PCR, VES, procalcitonin (PCT), D-dimer. The worsened picture was evaluated, considering organ dysfunction with the delta sequential organ failure assessment score (SOFA), in ranges from 0 to 24, and included points related to six organ systems: respiratory (hypoxemia), coagulatory (thrombocytopenia), liver (hyperbilirubinemia), cardiovascular system (hypotension), neurologic (low-level consciousness), and renal (oliguria or elevated creatinine).

### 2.2. CT Technique

Chest CT scans were performed at the time of hospital admission and during the hospital stay, with a 64-slice scanner (Toshiba Aquilion 64-Slice CT, Tokyo, Japan) dedicated to COVID-19 patients. CT examinations were performed with the patient in the supine position using a standard dose protocol, without contrast intravenous injection. The scanning range was from the apex to the base of the lungs. The tube voltage and the current tube were 120 kV and 100–200 mA, respectively. All images were obtained with a standard dose scanning protocol, reconstructed at 1.0 mm slice thickness, with 1 mm increment, 512 × 512 mm. Images were reconstructed with a sharp reconstruction kernel for parenchyma (FC13 on Toshiba). The lung window setting was at a window level of −600 Hounsfield units (HU) and window width of 1600 HU.

### 2.3. CT Post Processing

DICOM data were transferred into a PACS workstation and CT images were evaluated by two expert radiologists, in consensus, at the same reading session, using the clinically available computer tool Thoracic VCAR software (GE Healthcare, Chicago, IL, USA). The software provides automatic segmentation of the lungs and automatic segmentation and tracking of the airway tree. It provides the classification of voxels based on Hounsfield units and a color-coded display of the thresholds within a segmented region. Thoracic VCAR provided automatic segmentation of the lungs, and was performed using adaptive density based morphology. The lungs were extracted by using an optimal thresholding to identify low-density fields in the scans, region growing (automating seed generation method to segment an image into regions, with respect to a set of seeds) and void filling. The three-dimensional hole filling was used to fill the lung cavities created by the elimination of normal blood vessels during the thresholding process, while airways were automatically segmented and exempted by iterative application of increasingly restrictive constraints, to a thresholding and 3D region growing process. The software complies with the regulatory requirements of Council Directive 93/42/EEC concerning medical devices (CE 0459) and FDA regulations. Lung parenchyma was divided by Hounsfield unit (HU) intervals from−1024 to less than −977 HU, representing emphysematous changes [24]; values higher than −977 to −703 HU, representing normal parenchyma [25,26]; values from −703 to −368 HU, representing ground glass opacity (GGO); and values higher than −100 to 5 HU, representing consolidations [17,25,27,28]; the remaining lung parenchyma is classified as other. Thoracic VCAR software, representing the percentages of ground-glass opacity volume, consolidation volume, and emphysema volume in both lungs. Total lesion calculation was also performed, which made a total of ground-glass opacity and consolidation volumes [17]. The Thoracic VCAR is already in clinical practice in Chest CT affected by COVID-19 infections [17,29,30].

### 2.4. Statistical Analysis

Continuous data were expressed in terms of median value and range.

The Mann–Whitney test and Kruskal–Wallis test were used to assess statistically significant differences among groups. *p* value < 0.05 was considered significant for all tests.

All analyses were performed using Statistics Toolbox of MATLAB R2007a (The Math-Works Inc., Natick, MA, USA).

## 3. Results

A total of 341 CT examinations, including baseline and follow-up CTs, were analyzed. Thoracic VCAR software was unable to perform the quantification in 16/341 (4.7%) cases, both automatically and manually; therefore, the findings of 325 CTs were reported in the results. Among 140 enrolled patients, 11 patients died, while 129 patients were discharged after a median hospitalization period of 14 days (range, 4–50 days).

No statistically significant difference was found in the quantified volume distribution in the right and left lungs (*p* value > 0.23 at Mann–Whitney test).

**Figure 1:**
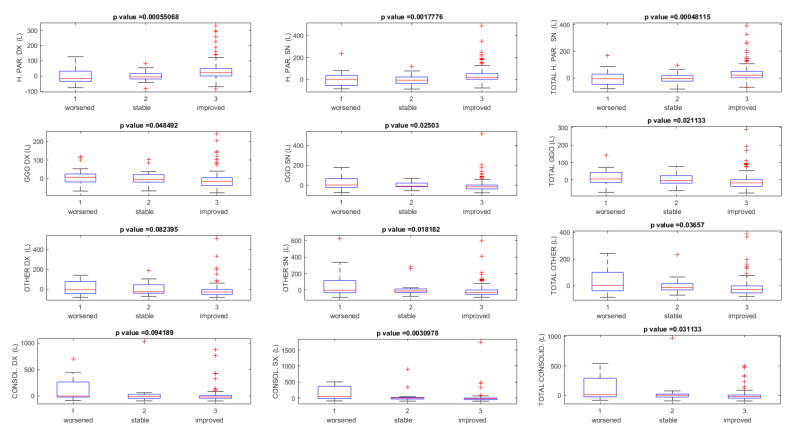
Boxplots of quantified volumes on CT using the computer tool based on the clinical condition (stable, improved, and worsened). Note: H. PAR. = healthy parenchyma, GGO = ground glass opacity; CONSOL = consolidation; R = right; L = left.

Table 1 reports the percentage changes on quantified volumes between baseline CT and follow-up CTs, grouping the patients based on their clinical conditions (stable condition, improved, and worsened condition). Statistically significant differences were found (*p* value ≤ 0.05 at Kruskal–Wallis test) among quantified volumes of healthy residual parenchyma, GGO, consolidation and total pulmonary volume, considering different clinical conditions (stable, worsened, improved) (see Figure 1).

Table 2 reports the quantified volumes at the last CT follow-up as percentage values of the total lung volumes, grouping the patients based on outcome in those dead and those discharged. CT was not performed on cadavers; the death was an outcome, which was retrospectively included, to differentiate findings of patients who survived vs. patients who died during the hospitalization.

**Figure 2:**
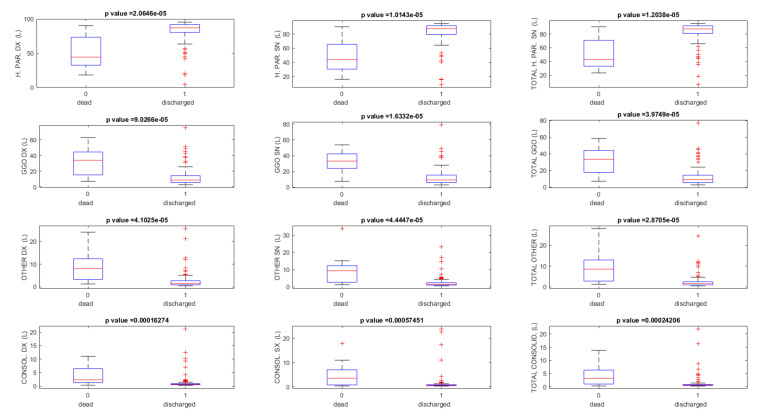
Boxplots of the quantified volume considering dead patients and discharged patients. Note: H. PAR. = healthy parenchyma, GGO = ground glass opacity; CONSOL = consolidation; R = right; L = left.

Statistically significant differences were also found (*p* value ≤ 0.05 at Kruskal–Wallis test), based on patients outcomes between dead patients and discharged patients, for quantified volumes of healthy residual parenchyma (42.9% versus 87.5%, retrospectively), of GGO (33.5% versus 9.0%, retrospectively), and of consolidation (3.2% versus 0.7%, retrospectively) (Figure 2). GGO and consolidation at the last follow-up, considering the discharged patients, had, as a median value, 0.37 and 0.03 L, respectively. Among discharged patients, a complete disease resolution of the CT scan was observed in 62/129 (48.1%) patients with a lung disease involvement ≤5%; a lung disease involvement from 5% to 15% was found in 40/129 (31.0%) patients, while 27/129 (20.9%) patients had lung disease involvement included, between 16 and 30%.

**Figure 3:**
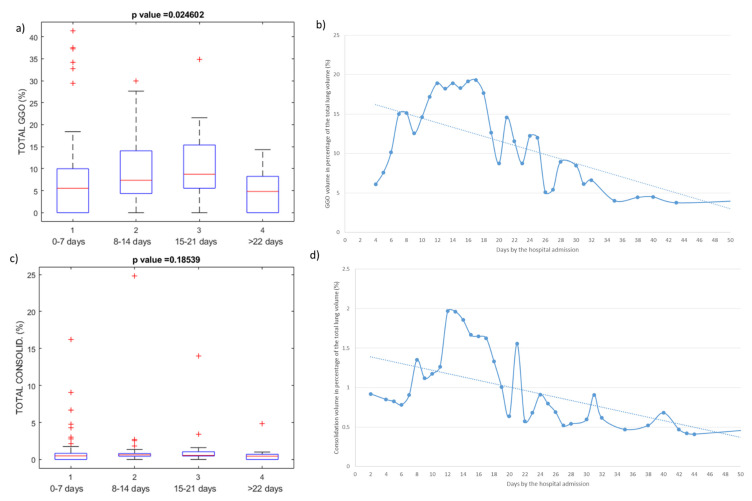
Temporal course of quantified volume on chest CT: in (**a**) and (**c**) boxplots of GGO volume and consolidation volume grouping the temporal course in 0–7 days (N. 25), 8–14 days (N. 50), 15–21 days (N. 37), and >22 days (N. 28); in (**b**) and (**d**) the temporal course of GGO and consolidation volume as a percentage value of total lung volume.

In Figure 3, we reported the evolution of the quantified GGO and consolidation volumes calculated on chest CT. Figure 3a,c shows the boxplots of GGO volume and consolidation volume, grouping the temporal course in 0–7 days, 8–14 days, 15–21 days, and ≥22 days after hospital admission. Exclusively GGO volume presented statistically significant differences among these groups. Considering Figure 3b,d, we can observe that GGO volume increased until the 16 days and consolidation volume until the 12 days, 8–21 days is the advanced period with the most severe lung involvement; after the extent of involvement started to decrease, particularly, after 21 days, the absorption was more obvious.

**Figure 4:**
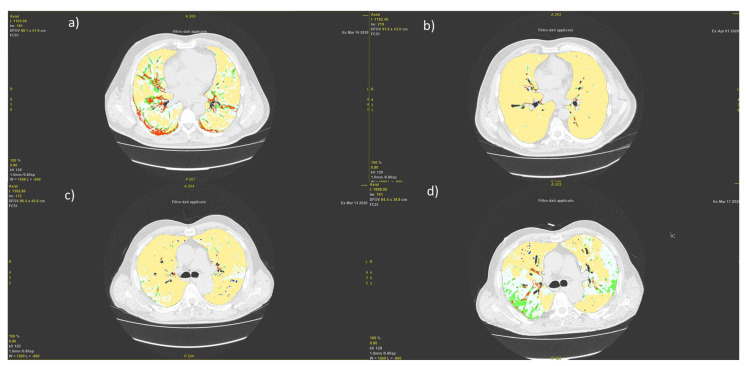
Representative cases of a patient with a CT panel improved and then discharged (**a**) and (**b**) (at baseline and follow-up CT), and of a case of a patient with a CT panel worsened and then died (**c**) and (**d**) (at baseline and follow -up CT).

Figure 4 showed two representative cases: a patient with a CT panel improved and then discharged Figure 4a,b and a case of a patient with a CT panel worsened and then died Figure 4c,d.

## 4. Discussion

Several previous studies [6,7,8,9,10,11,12,13,14,15,16] have described the main CT signs of COVID-19, summarized as GGO, crazy-paving pattern, and consolidation. Several methods of disease extent quantification at chest CT using machine learning and AI tools have been proposed, including the extent of emphysema, GGO, and consolidation [31,32,33,34,35,36,37,38,39,40,41,42,43,44,45,46,47,48,49,50,51]. Few studies have investigated the changes in CT findings associated with COVID-19 pneumonia in the follow-up, quantifying the evolution and the absorption of the abnormalities visible on CT using a computer automatic aided tool.

Zhou et al. [36] investigated CT images of 100 confirmed COVID-19 pneumonia patients, to describe the lesion distribution, CT signs, and evolution during different courses. They reported that the course of COVID-19 pneumonia consists of three stages: 1–7 days is the early rapid progressive stage, 8–14 days is the advanced stage, and after 14 days, the abnormalities start to decrease. In the early rapid progressive stage, GGO plus a reticular pattern, GGO plus consolidation, and GGO, were all common signs; in the advanced stage, signs of progression and absorption coexisted; lung abnormalities showed an asynchronous process, with parts with absorption and parts progressing. Lung abnormalities predominantly showed peripheral, middle, and lower distribution.

Pan et al. [5] assessed the chest CT to determine the changes in the findings associated with COVID-19 from initial diagnosis until patient recovery. They reported that lung abnormalities on chest CT scans showed the greatest severity approximately 10 days after the initial onset of symptoms.

Wang at al [50] reported the analysis on 366 CT scans to assess the temporal changes of CT findings in 90 patients with COVID-19 pneumonia. Their results showed that CT findings progressed rapidly, and peaked during illness days 6–11. The predominant pattern of abnormalities after symptom onset was ground-glass opacity. The percentage of mixed patterns peaked on illness days 12–17, and became the second most predominant pattern thereafter. Pure ground-glass opacity was the most prevalent subtype of ground-glass opacity after symptom onset. The percentage of ground-glass opacity with irregular linear opacity peaked on illness days 6–11 and became the second most prevalent subtype thereafter. The distribution of lesions was predominantly bilateral and subpleural. Sixty-six of the 70 patients discharged (94%) had residual disease on final CT scans.

However, to the best of our knowledge, there is no study in the literature reporting on the temporal changes of CT findings, using an automatic tool to quantify the abnormality in lung parenchyma (due to COVID-19 pneumonia).

According to the recent literature, we reported that GGO is the most representative sign of COVID-19 disease on chest CT, and that statistically significant differences were found among quantified volumes of healthy residual parenchyma, GGO, consolidation and total pulmonary volume, considering different clinical conditions (stable, improved, and worsened). Statistically significant differences were also found, based on patient outcomes between dead patients and discharged patients, for quantified volumes of healthy residual parenchyma (42.9% versus 87.5%, retrospectively), of GGO (33.5% versus 9.0%, retrospectively), and of consolidation (3.2% versus 0.7%, retrospectively). We reported that, among discharged patients, a complete disease resolution on CT scans was observed in 62/129 patients with lung disease involvement ≤5; lung disease involvement ranging from 5% to 15% was found in 40/129 patients, while 27/129 (20.9%) patients had lung disease involvement, between 16 and 30%. The discharged patients at the last follow-up had a percentage change of lung disease involvement of 12.5% while the dead patients of 57.1%.

Moreover, we demonstrated that GGO and consolidation at the last follow-up were almost completely absorbed, and that 8–21 days of hospital admission was the advanced period with the most severe lung involvement. After 16 days of hospital admission, the abnormalities identified by chest CTs started to improve and, in particular, after 21 days, the absorption was more obvious.

In this study, we reported that no statistically significant difference was found in the quantified volume distribution in the right and left lung—in contrast to what was reported by Li et al. [52] and Nagra et al. [53]. Li et al. [52] noticed a side-preference of lung lesions in COVID-19. The lesions in the right lungs were significantly larger and developed faster than those on the left. Moreover, the level of the right-over-left preference of lung injury was significantly correlated with the potential need for intensive care and inpatient mortality. Nagra et al. [53] concluded that in COVID-19 the right lung has a higher degree of opacification on a plain radiograph than the left lung.

We believe that analysis of CT findings, using a computer tool based on different thresholding Hounsfield unit settings, could identify pulmonary abnormalities and lung recruitment, and we believe that knowledge of the percentage of potentially recruitable lung evolution may be important to establish the therapeutic efficacy in COVID-19 disease.

There are still some limitations in this study. First, the time for CT re-examination of each patient is not standardized. Second, the retrospective and monocentric nature of the study. Third, the absence of laboratory findings to correlate with the CT results.

## 5. Conclusions

In conclusion, we reported that CT findings, using a computer automatic tool based on different thresholding Hounsfield Unit settings, could identify pulmonary abnormalities and lung recruitment. Moreover, we demonstrated that discharged patients had lung disease involvement of 12.5%, while for dead patients it was 57.1%; a complete disease resolution on chest CT scans was observed in 48.1% of patients using a computer aided tool to quantify the GGO and consolidation volumes. Moreover, 8–21 days of hospital admission is the advanced stage, with peak levels of abnormalities on CTs; after 16 days, the abnormalities started to improve. Therefore, CT has proven to be a useful tool in following the evolution of the disease, by clarifying the progression/regression timing of the disease.

## Figures and Tables

**Table 1:** Percentage change of quantified volume between baseline CT and follow-up CTs.

		H. PAR. R (%)	H. PAR. L (%)	TOTAL H. PAR. (%)	GGO R (%)	GGO L (%)	TOTAL GGO (%)	OTHER R (%)	OTHER L (%)	TOTAL OTHER (%)	CONSOL. R (%)	CONSOL. L (%)	TOTAL CONSOLID. (%)	TOTAL LUNG VOL. R (%)	TOTAL LUNG VOL. L (%)	TOTAL LUNG VOL (%)
Worsened (N. 10)	Median value	−23.32	−11.87	−18.32	21.19	50.23	42.29	32.42	12.59	22.90	45.88	54.41	37.61	−6.70	−5.56	−3.50
	Minimum value	−76.59	−83.42	−79.78	−67.78	−68.76	−68.21	−71.78	−74.55	−71.02	−86.73	−83.40	−82.67	−49.03	−60.31	−54.36
	Maximum value	101.71	81.72	85.30	139.50	207.06	168.57	200.23	628.14	242.41	694.60	507.97	543.48	43.43	41.13	41.64
Stable (N. 11)	Median value	−15.10	−11.11	−12.87	5.92	−2.52	2.20	−12.24	−5.45	−7.25	−9.78	−0.94	3.34	−5.34	−5.85	−8.06
	Minimum value	−85.69	−85.58	−82.97	−66.78	−56.24	−62.03	−74.14	−76.63	−71.11	−93.67	−93.98	−93.84	−47.81	−46.60	−47.26
	Maximum value	115.23	185.07	95.99	203.05	517.48	289.99	334.34	412.35	365.69	1030.43	900.34	971.51	69.84	397.22	142.59
Improved (N. 119)	Median value	23.99	22.26	23.17	−19.21	−17.66	−17.78	−32.71	−29.74	−31.09	−20.80	−18.31	−21.63	10.00	13.20	13.32
	Minimum value	−45.43	−72.40	−52.02	−77.15	−77.58	−75.48	−83.24	−89.89	−87.02	−96.29	−96.07	−96.22	−33.14	−66.22	−38.05
	Maximum value	329.90	488.56	389.44	240.24	180.80	192.19	516.14	592.85	388.46	872.45	1756.37	500.33	147.37	192.86	165.33
Total (N. 140)	Median value	15.21	18.93	17.01	−10.11	−15.02	−13.97	−26.81	−23.56	−26.12	−18.99	−11.82	−14.10	7.25	10.81	8.56
	Minimum value	−85.69	−85.58	−82.97	−77.15	−77.58	−75.48	−83.24	−89.89	−87.02	−96.29	−96.07	−96.22	−49.03	−66.22	−54.36
	Maximum value	329.90	488.56	389.44	240.24	517.48	289.99	516.14	628.14	388.46	1030.43	1756.37	971.51	147.37	397.22	165.33

Note: H. PAR. = healthy parenchyma, GGO = ground glass opacity; CONSOL = consolidation; TOTAL LUN VOL. = total lung volume; R= right; L = left; Lung parenchyma was divided by Hounsfield unit (HU) intervals from −1024 HU to less than −977 HU representing emphysematous changes, values higher than −977 HU to −703 HU representing normal parenchyma, values from −703 HU to −368 HU representing ground glass opacity (GGO) and values higher than −100 HU to 5 HU representing consolidations; the remaining lung parenchyma is classified as other.

**Table 2:** Quantified volumes on CT at the last follow-up.

		H. PAR. R (%)	H. PAR. L (%)	TOTAL H. PAR. L (%)	GGO R (%)	GGO L (%)	TOTAL GGO (%)	OTHER R (%)	OTHER L (%)	TOTAL OTHER (%)	CONSOL. R (%)	CONSOL. L (%)	TOTAL CONSOLID. (%)
Dead (N. 11)	Median value	44.46	44.06	42.89	34.01	33.12	33.58	8.09	9.30	8.55	2.34	3.55	3.21
	Minimum value	18.34	15.94	23.36	7.03	7.30	7.15	1.31	1.20	1.26	0.32	0.33	0.32
	Maximum value	90.72	90.40	90.58	63.00	53.70	58.55	24.08	34.00	28.12	11.05	17.92	13.78
Discharged (N. 129)	Median value	87.36	87.71	87.48	8.67	9.19	9.02	1.57	1.51	1.56	0.69	0.61	0.65
	Minimum value	4.55	8.64	6.41	2.73	2.71	2.72	0.51	0.56	0.54	0.27	0.30	0.29
	Maximum value	95.76	95.14	95.45	75.17	79.12	76.99	25.65	23.37	24.61	21.28	23.94	21.95
Total (N. 140)	Median value	86.67	87.10	86.31	9.77	9.96	9.96	1.61	1.61	1.69	0.72	0.65	0.69
	Minimum value	4.55	8.64	6.41	2.73	2.71	2.72	0.51	0.56	0.54	0.27	0.30	0.29
	Maximum value	95.76	95.14	95.45	75.17	79.12	76.99	25.65	34.00	28.12	21.28	23.94	21.95

Note: H. PAR. = healthy parenchyma, GGO = ground glass opacity; CONSOL = consolidation; TOTAL LUN VOL. = total lung volume; R= right; L = left; Lung parenchyma was divided by Hounsfield unit (HU) intervals from −1024 HU to less than −977 HU representing emphysematous changes, values higher than −977 HU to −703 HU representing normal parenchyma, values from −703 HU to −368 HU representing ground glass opacity (GGO) and values higher than −100 HU to 5 HU representing consolidations; the remaining lung parenchyma is classified as other.

## Data Availability

All data are reported in the manuscript.
